# Progress in the Elimination of Organic Contaminants in Wastewater by Activation Persulfate over Iron-Based Metal–Organic Frameworks

**DOI:** 10.3390/nano14050473

**Published:** 2024-03-05

**Authors:** Keke Zhi, Jiajun Xu, Shi Li, Lingjie Luo, Dong Liu, Zhe Li, Lianghui Guo, Junwei Hou

**Affiliations:** 1 Department of Engineering, China University of Petroleum-Beijing at Karamay, Karamay 834000, China; zhikeke@cupk.edu.cn (K.Z.); 2022016312@st.cupk.edu.cn (J.X.); 2022015893@st.cupk.edu.cn (S.L.); 2022015729@st.cupk.edu.cn (L.L.); 2023216893@st.cupk.edu.cn (D.L.); 2 State Key Laboratory, Heavy Oil Processing-Karamay Branch, Karamay 834000, China; 2020015113@st.cupk.edu.cn; 3 Department of Petroleum, China University of Petroleum-Beijing at Karamay, Karamay 834000, China

**Keywords:** iron-based MOFs, activation persulfate, advanced oxidation process, organic contaminants

## Abstract

The release of organic contaminants has grown to be a major environmental concern and a threat to the ecology of water bodies. Persulfate-based Advanced Oxidation Technology (PAOT) is effective at eliminating hazardous pollutants and has an extensive spectrum of applications. Iron-based metal–organic frameworks (Fe-MOFs) and their derivatives have exhibited great advantages in activating persulfate for wastewater treatment. In this article, we provide a comprehensive review of recent research progress on the significant potential of Fe-MOFs for removing antibiotics, organic dyes, phenols, and other contaminants from aqueous environments. Firstly, multiple approaches for preparing Fe-MOFs, including the MIL and ZIF series were introduced. Subsequently, removal performance of pollutants such as antibiotics of sulfonamides and tetracyclines (TC), organic dyes of rhodamine B (RhB) and acid orange 7 (AO7), phenols of phenol and bisphenol A (BPA) by various Fe-MOFs was compared. Finally, different degradation mechanisms, encompassing free radical degradation pathways and non-free radical degradation pathways were elucidated. This review explores the synthesis methods of Fe-MOFs and their application in removing organic pollutants from water bodies, providing insights for further refining the preparation of Fe-MOFs.

## 1. Introduction

With the rapid development of modern industry, wastewater treatment has become an urgent environmental issue. Many countries have issued policies related to wastewater. China has proposed the following principle for sewage treatment, “priority should be given to water conservation, measures should be adapted to local conditions, and policies should be implemented by category”. As early as the 20th century, the UK passed legislation to strictly control the discharge of pollutants and increased research and utilization of new technologies for wastewater treatment. Germany has taken measures such as “sewage elevators” and green embankments to repair rivers and treat wastewater.

Wastewater can be mainly divided into antibiotic pollutant wastewater, organic dye wastewater, phenolic plasticizers, composite pollutants, etc. Due to their different compositions, the catalysts used to degrade wastewater are not quite the same [1]. Antibiotics are rampant in water bodies due to high consumer demand and inappropriate use of antibiotics, as well as inadequate technologies for the control of and reduction in antibiotic-containing wastes [2]. In aquatic ecosystems, the presence of antibiotics and their degradation products may pose a threat to non-target organisms at different trophic levels, including bacteria, algae, plants, invertebrates, and fish [3]. Synthetic organic dyes are one of the most harmful sources of pollution [4]. Due to their low price and high chemical stability, they are widely used in the paper, tanning, pharmaceutical, photographic, and cosmetic industries. Unfortunately, dyes are highly toxic and can have carcinogenic and mutagenic effects on living organisms even at very low concentrations [5]. Removal of dyes from wastewater has become one of the present concerns. Phenolic pollutants are also a very harmful source of pollution to the environment. As an example, BPA is widely used in beverage and food containers, adhesives, flame retardants, and building materials. The widespread use of BPA has led to its continuous release and distribution into the aquatic environment [6]. According to the U.S. Environmental Protection Agency, BPA is regularly released into the environment at a rate of about 106 pounds per year. Additionally, BPA is considered a typical endocrine disruptor with estrogenic effects and significant biological toxicity [7]. It has been shown to have estrogenic activity even at concentrations below 1 mg/L [8]. Therefore, there is a great demand for effective treatment technology for BPA in water.

At present, the following methods are mainly applied to the degradation and disposal of organic pollutants: (1) The adsorption method [9,10,11]. Adsorption is the process of accumulating particulates from one phase (liquid or vapor) onto the surface of a solid substrate (adsorbent). The main advantages of the adsorption method include a simple operation, low cost, high efficiency, and the absence of toxic by-products. The efficiency of the adsorption process depends on the type of adsorbent and factors such as the specific surface area, pore size, and porosity of the adsorbent [11]. The adsorption process is influenced by parameters such as the solution pH, contact time, initial concentration of contaminants, and sample intensity [10]. (2) Advanced Oxidation Technology (AOT). In conjunction with the adsorption process, AOT can generate various reactive oxygen species, including sulfate radicals (SO_4_^•−^ [12]), hydroxyl radicals (•OH [13]), and singlet oxygen (^1^O_2_ [14]). Due to the high redox potential of reactive oxygen species, the strong oxidation power generated can be utilized to gradually reduce and decompose antibiotic organic pollutants into small molecules, ultimately achieving complete mineralization under mild reaction conditions. Currently, advanced oxidation methods include electrochemical advanced oxidation [15], Fenton advanced oxidation [16], photocatalysis [17] and advanced oxidation [18].

It is worth mentioning that persulfate-based Advanced Oxidation Technology (PAOT) is considered one of the most promising technologies due to its fast reaction speed (φ^ϑ^ = 2.58 V) and strong removal ability. This is attracting more and more attention [19]. There are two types of persulfates (PS), permonosulfate and perdisulfate. The premise for the oxidizing effect of persulfate is that it needs to be activated. When persulfate is activated by catalysts, it produces reactive oxygen species that attack stubborn organic pollutants in the water environment. Various iron-based catalyst materials exist, such as Fe^2+^ [20], zero-valent ferrum [21], and ferrum oxides [22]. Because of their low cost, high efficiency, and large reserves, they are used for PS activation.

MOFs, with their high porosity, large specific surface area, and high metal ion density are ideal materials for catalysis. MOFs are a type of hollow coordination polymers formed by metal and organic coordination, possessing both metal activity and organic ligand flexibility, and are widely used in separation and energy storage, among other fields [23,24,25]. As early as the 1990s, Li et al. [26] synthesized MOFs with Zn^2+^ as the central atom and applied them to the field of gas storage. Since then, many scientists have begun to study this material. In the 21st century, Gerard Ferey’s team [27] synthesized MIL-100 and MIL-101, and since then, more MOFs have been synthesized for multiple pathways. However, in the early stage, people mostly used their stability and gas adsorption properties for gas storage. As more and more MOF materials were synthesized, other properties of MOFs were gradually discovered, and over 2000 types have been synthesized and utilized [28].

Iron-based catalysts are widely used in the catalysis of PS due to their low price, excellent catalytic efficiency, and huge storage capacity. There are a variety of Fe-MOFs, such as MIL-53(Fe), MIL-88(Fe), etc. Fe-MOFs formed by oxygen-containing organic ligands and iron ions are more suitable for wastewater treatment because Fe-O clusters are excited to produce more active substances, giving them a strong catalytic activity, ease of preparation, and robust stability. Currently, there are two primary mechanisms for the degradation of organic contaminants of Fe-based MOFs including the non-radical pathway and the radical pathway. Free radicals mainly include SO_4_^•−^ and •OH. The main mechanisms of non-free radicals are ^1^O_2_ and electron transfer [29].

Currently, most of the mainstream MOFs’ reviews are comprehensive, with few focusing on single metals. Our review aims to follow this trend and fill the gap in this area. Furthermore, reviews that systematically describe and categorize Fe-based MOFs and their applications in activation persulfate for the elimination of organic contaminants in wastewater have also rarely been mentioned. In this review, we describe the preparation of different types of Fe-MOFs using hydrothermal, microwave-assisted, and dry gelation methods. Additionally, different modifications of Fe-MOFs, such as metal doping, functional material doping, and molecularly imprinted techniques, are also presented. Meanwhile, this paper reviews the performance of Fe-MOFs in removing pollutants such as sulfonamides, tetracyclines, organic dyes, and phenols from water, along with their mechanisms. We believe that systematically reviewing the progress of Fe-MOFs in treating water pollution and understanding its mechanisms is of great significance.

## 2. Preparation and Modification of MOFs Catalysts

Fe-MOFs, an important branch of MOFs, not only possess characteristics like porous channels, an extremely high specific surface area, and multiple active sites similar to other MOFs but also are more environmentally friendly. Due to these advantages, Fe-MOFs are more suitable for environmental governance. There are many types of Fe-MOFs, such as MIL-Fe (Material of Institute Lavoisier) [30], ZIF-Fe (Zeolitic Imidazolate Framework) [31], BTC (Bimetallic), UIO (University of Oslo) [32], etc.

### 2.1. Synthesis of Fe-MOFs

MOFs are crystalline porous solid materials formed by coordination bonds between organic linkers and metal ions or clusters. Using this bottom-up synthesis approach, the chemical moieties within the framework can be spatially controlled [33,34].

**Figure 1:**
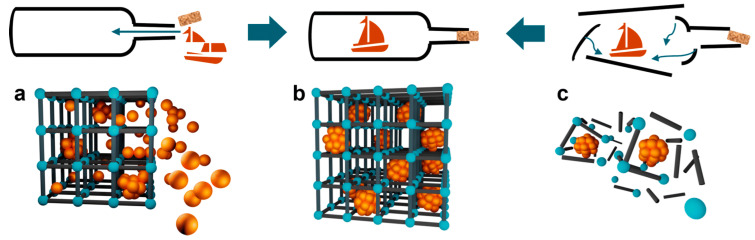
The overview of strategies to synthesize MOF catalysts [36]. (**a**) ‘ship-in-bottle’ and (**c**) ‘bottle-around-ship’ approaches both yielding (**b**). Adapted with permission from Ref. [36]. Copyright 2022, copyright Kathrin L. Kollmannsberger.

MOFs are constructed from metal nodes, ions or clusters, and organic linkers, assembled into crystalline and highly porous frameworks [35]. The “ship-in-bottle” approach (pore-restricted growth) is a synthesis strategy in which metal aggregates are strategically constructed within MOF pores from the “bottom up” (e.g., Figure 1). The components used for this method must be able to penetrate the MOF pores, similar to constructing a model ship inside a glass bottle. To incorporate nanoscale metal aggregates into MOFs, it is critical to use precursors that inherit the appropriate reactivity so that they can be processed in synthesis while still having sufficient reactivity to be converted into the final nanoscale metal aggregates after loading the precursor into the pore system [36].

#### 2.1.1. Synthesis of MIL-Fe

**Figure 2:**
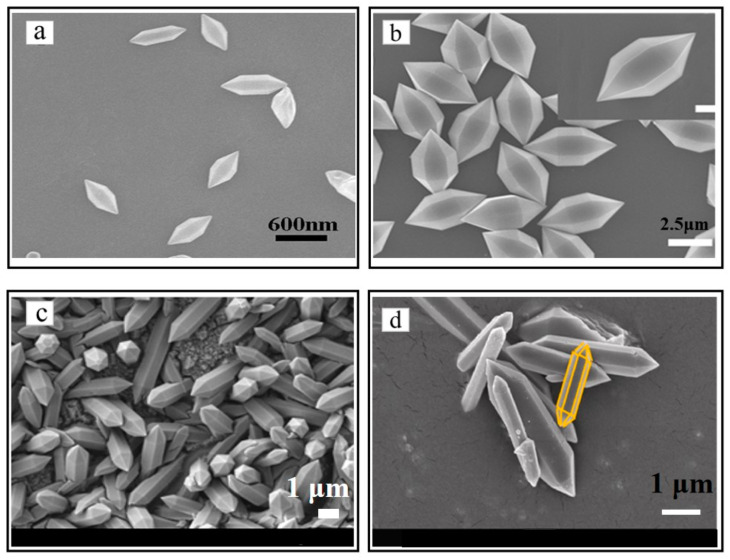
The SEM of MIL-53(Fe) [43] (**a**), MIL-88(Fe) [44] (**b**), MIL-101(Fe) [45] (**c**), MIL-100 (Fe) [46] (**d**). Adapted with permission from Ref. [43]. Copyright 2017, copy-right Xuechuan Gao. Adapted with permission from Ref. [44]. Copyright 2016, copy-right Xuechao Cai. Adapted with permission from Ref. [45]. Copyright 2024, copy-right Abhijit Das.

With a large specific surface area, abundant active sites, flexible pore structure, and excellent water stability, MILs have attracted increasing attention as effective environmental catalysts [37]. The current mainstream MIL-Fe catalysts include MIL-53(Fe) [38,39], MIL-88(Fe) [40], MIL-100(Fe) [41,42], MIL-101(Fe) [6,12], etc. MIL-53(Fe) is based on Fe(III) as a metal ion and terephthalic diformic acid is a hexagonal pyramid-shaped crystal of organic ligands [43]. The SEM image is shown below Figure 2a. MIL-88(Fe) is based on Fe(III) as a metal ion and dicarboxylic acid and its derivatives are hexagonal microrod-like crystals of organic ligands [44]. The SEM image is shown below Figure 2b. MIL-100(Fe) is based on Fe(III) as a metal ion and homobenzene tricarboxylic acid and its derivatives are octahedral crystals prepared from organic ligands [45]. The SEM image is shown below Figure 2d. MIL-101(Fe) is based on Fe(III) as a metal ion and dibasic carboxylic acid and its derivatives are regular polyhedron crystals prepared from organic ligands [46]. The SEM image is shown below Figure 2c.

MILs are typically prepared according to groundbreaking synthesis procedures, and various modifications are made to synthesis conditions, such as time, temperature, pH value, additives, and carrier materials [47]. This paper summarizes the preparation of MILs by hydrothermal, microwave synthesis, and dry gel conversion methods.

Our review has summarized a portion of the common synthesis methods for MIL-Fe in Table 1. In this case, hydrothermal method is one of the most common, most used and most widely applied methods. In addition, for the shortcomings of the hydrothermal method a wide range of scholars have improved and proposed other methods, such as microwave-assisted method, dry gel method and so on.

##### Hydrothermal Method/Solvothermal Method

Hydrothermal synthesis is a process in which crystalline powders, coatings, and single crystals can be obtained directly from a solution at relatively low temperatures. It enables excellent powder properties such as high purity, phase stability (stoichiometry), controlled particle size, narrow particle size distribution, and controlled morphology. Therefore, hydrothermal synthesis can help researchers/manufacturers overcome some technical challenges and assist them in developing new technologies for sustainable growth [64].

Farzaneh Mahmoud et al. [65] used 1,3,5-benzenetricarboxylic acid (H_3_-BTC), ferric nitrate nonahydrate (Fe(NO_3_)_3_·9H_2_O), tetraethyl orthosilicate (TEOS), hydrochloric acid, ethanol, and sodium hydroxide to make MIL-100(Fe). Ferric nitrate nonahydrate (Fe(NO_3_)_3_·9H_2_O) was dissolved in distilled water, then 1,3,5-benzenetricarboxylic acid was added to the above solution while stirring for 30 min. The mixture was completely transferred to a Teflon-liner autoclave and heated at 160 °C. After cooling the autoclave, the dark orange precipitate was collected with a centrifuge and washed with a two-step purification process to remove any unreacted species. The precipitate was washed with distilled water and ethanol, stirred at 65–70 °C for 3 h, collected, and dried at room temperature.

**Figure 3:**
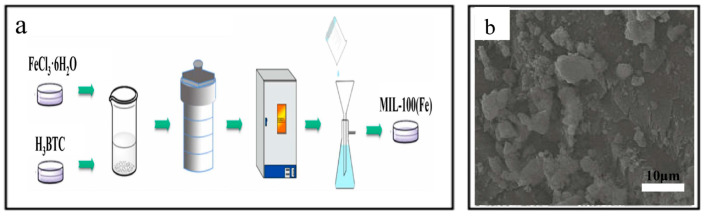
(**a**) The schematic diagram for MIL-100(Fe) synthesis hydrothermal method. (**b**) The SEM of MIL-100(Fe) [42]. Adapted with permission from Ref. [42]. Copyright 2024, copy-right Huizhong Zhao.

Zhao et al. [42] synthesized MIL-100(Fe) following the steps in Figure 3a. FeCl_3_·6H_2_O, H_3_BTC, and deionized water were mixed and stirred magnetically for 60 min at room temperature. The stirred solution was then transferred into the reactor, sealed, and crystallized at 150 °C for 12 h. After natural cooling, the reactor was opened, and the product was dried after filtration. The reaction products were alternatively washed. The SEM image in Figure 3b reveals a smooth and flat surface structure. Additionally, the arrangement of MIL-100(Fe) appeared regular, with some irregular bulk particles observed.

##### Microwave-Assisted Synthesis

Microwave-assisted synthesis has significant advantages in saving time and energy compared to hydrothermal methods. Additionally, this technology allows for the production of monodisperse nanocrystalline particles, which are more wear-resistant due to their small size [66].

**Figure 4:**
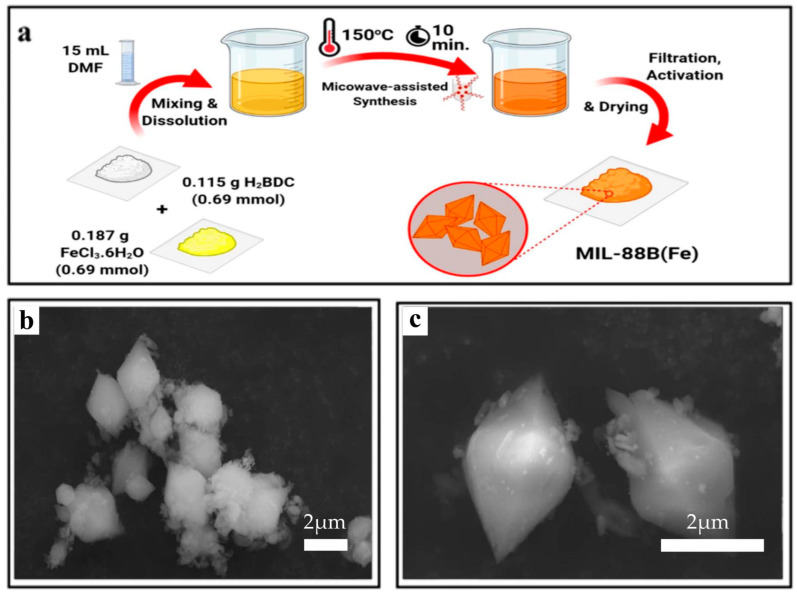
(**a**) The schematic diagram for MIL synthesis procedures via microwave-assisted technique. (**b**,**c**) The SEM of MIL-88(Fe) [67]. Adapted with permission from Ref. [67]. Copyright 2022, copy-right Mahmoud Y. Zorainy.

Zorainy et al. [67] discovered a new and easier way to synthesis MIL-Fe. As in the typical procedure, as illustrated in Figure 4a, a molar mixture of FeCl_3_·H_2_O and H_2_BDC was directly weighed into the reaction vial. Subsequently, 15 mL of DMF was added to the mixture, and the solution was magnetically stirred for 30 min at room temperature. Afterward, the vial was placed into the reaction chamber of the microwave reactor. The reaction proceeded after heating the reaction medium to 150 °C and maintaining the temperature for 10 min under constant stirring. Scanning electron microscopy was conducted to study the morphological characteristics of MIL-100(Fe). The SEM images of MIL-100(Fe) are shown in Figure 4b,c, from which it can be observed that most of the samples were amorphous aggregates.

##### Dry-Gel Conversion (DGC) Technology

The synthesis of porous materials with dry-gel transfer has potential advantages, including minimal waste disposal, a reduced reactor size, a reduced consumption of templates, and the possibility of continuous production. Additionally, DGC can be used to produce monolithic or shape-controlled porous materials from preformed gels [60].

**Figure 5:**
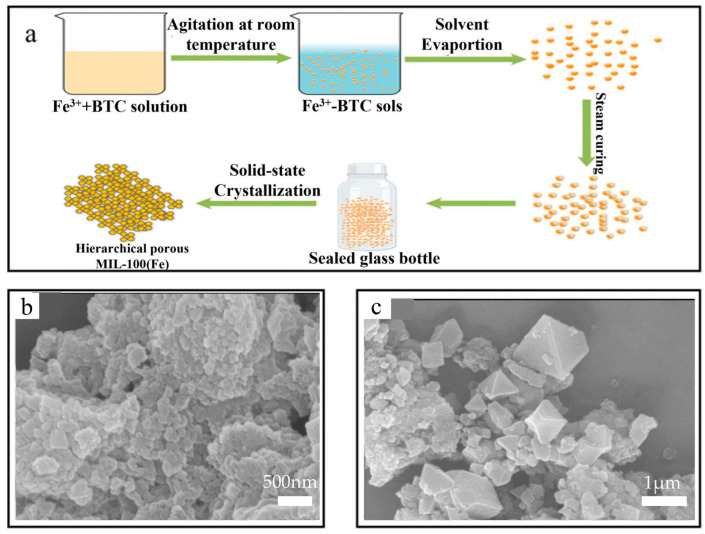
(**a**) The schematic diagram of catalysis synthesis by DGC. (**b**) The SEM of MIL-100(Fe)-DGC. (**c**) The SEM of MIL-100(Fe) [41]. Adapted with permission from Ref. [41]. Copyright 2019, copy-right Yanshu Luo.

Luo et al. [41] used an Fe(NO_3_)_3_·9H_2_O aqueous solution, and then H_3_BTC was added to the solution as shown in Figure 5a. The obtained light orange Fe-BTC sol, under strong agitation at room temperature for 3 h, was left in a freeze-drying device overnight to evaporate water. Then, the dry gels were ground into powders and placed in a humidity chamber for steam curing at room temperature. Scanning electron microscopy was conducted to study the morphological characteristics of MIL-100(Fe). The SEM images of MIL-100(Fe) are shown in Figure 5b,c, from which it can be observed that most of the samples were amorphous aggregates.

#### 2.1.2. Synthesis of ZIF-Fe

ZIFs is formed by 2-methylimidazole and Zn^2+^ nodes, which are porous and hydrophilic MOFs [68]. ZIFs, being a representative member of the MOF family, have garnered attention due to their chemical robustness, thermal stability, large surface area, and intersecting three-dimensional structural features [69].

The metal source of ZIFs is transition-metal ions such as Zn^2+^ [70]. Moreover, the organic linker is imidazole or imidazole derivatives (e.g., 2-methylimidazole), which, similar to the ligand, form a metal–imidazole–metal structure, akin to the Si-O-Si bond in traditional silica-based zeolites [71].

##### Hydrothermal Method

**Figure 6:**
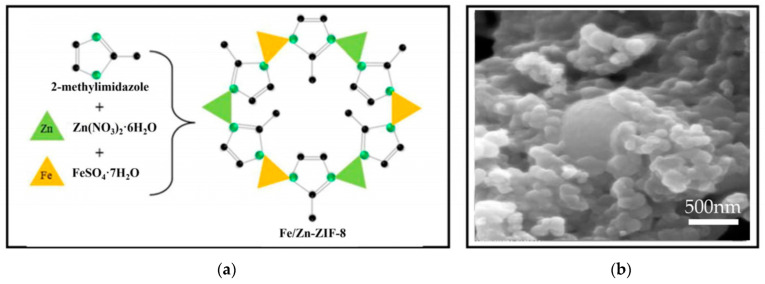
(**a**) The schematic diagram of catalysis synthesis by hydrothermal method. (**b**). The SEM of Fe-ZIF-8 [72]. Adapted with permission from Ref. [72]. Copyright 2023, copy-right Entisar M. Khudhair.

Khudhair et al. [72] dissolved Zn(NO_3_)_2_·6H_2_O and FeSO_4_·7H_2_O in methanol. A solution was made by mixing 2-methylimidazole and methanol, and then the solution was added to the Fe (II)-Zn (II) solution and stirred at ambient temperature. The white product was collected through centrifugation. Finally, after washing and drying, Fe-ZIF-8 was obtained. The brief synthesis process is shown in Figure 6a. With the addition of Fe, the structure of ZIF-8 became irregular and distorted, as shown in Figure 6b.

We have summarized the advantages and disadvantages of hydrothermal, microwave-assisted and dry gelation methods after certain literature research and presented them in the form of Table 2.

Overall, hydrothermal method is the most used and the earliest method for the preparation of Fe-MOFs. Although the hydrothermal method has the disadvantages of complicated reaction conditions, a high danger, and low reaction efficiency, the hydrothermal method has a wide range of uses, is the most mature technology, and has been widely used by many scholars. The microwave-assisted method and dry-gel method are both improved on the basis of the hydrothermal method and possess many limitations. It is undeniable that these two methods provide a synthesis method for some special materials and contribute to the development of Fe-MOFs. They effectively promote the reaction rate, improve the safety of the reaction process, and increase the purity and yield of the products. The dry-gelation method uses pressurized water vapor to convert a dry precursor gel into a product, which can significantly reduce the amount of water required to synthesize the product. Dry-gel transformation is an effective method for synthesizing zeolites. This method has many advantages such as a low water consumption, high yield, and being a simple procedure. It is widely used in molecular sieve synthesis.

### 2.2. Modification of Fe-MOFs

Fe-MOFs are materials with high adjustability and multifunctionality, and their application potential is extensive. Modification methods of Fe-MOFs can further improve their performance, enhance their adaptability, and promote their application in various fields. Some common Fe-MOFs modification methods include ionic doping modification, combining functional modification, molecular imprinting modification, etc.

#### 2.2.1. Ionic Doping

Covalent postsynthesis modification of Fe-MOFs with ionic liquids is an effective method to improve the stability of Fe-MOFs and provides the possibility of stabilizing metals and nanoparticles [73]. The introduction of metal nanoparticles in these structures drives the creation of bimetallic systems, which brings the synergistic effect of the metal nanoparticles introduced with the metal nodes in the Fe-MOF structure and enhances the catalytic activity [74].

**Figure 7:**
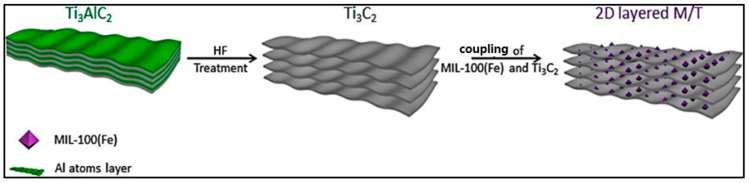
The schematic diagram of the preparation of MIL-100(Fe)/Ti_3_C_2_ [75]. Adapted with permission from Ref. [75]. Copyright 2019, copy-right Hanmei Wang.

Wang et al. [75] prepared MIL-100(Fe)/Ti_3_C_2_ by a facile synthetic method, as shown in Figure 7. Mil-100(Fe) and Ti_3_C_2_ Mxene were added into ethanol. Then, the mixture was stirred, ultrasonically treated, and dried. The product was then heated, and finally, the Ti_3_C_2_ Mxene nanosheets were coupled with MIL-100(Fe).

**Figure 8:**
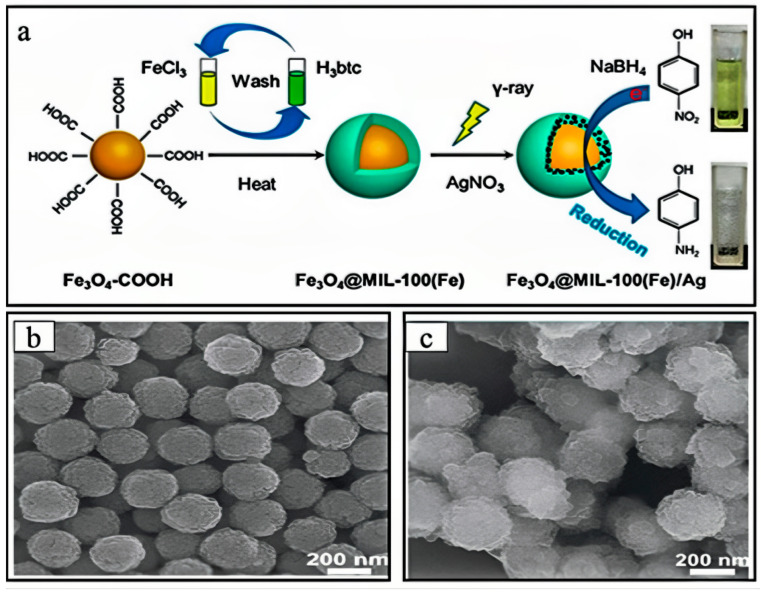
(**a**) The fabrication strategy of Fe_3_O_4_@MIL-100(Fe)/Ag nanocomposites. (**b**) The SEM of Fe_3_O_4_. (**c**) The SEM of Fe_3_O_4_ [76]. Adapted with permission from Ref. [76]. Copyright 2020, copy-right Shuquan Chang.

Chang et al. [76] activated the MOF particles under vacuum overnight. The detailed preparation process is shown in Figure 8a. The magnetic MOF particles were suspended in water and ultrasonically dispersed for 20 min until they became homogeneous. A quantity of AgNO_3_ solution was dropwise added to the above suspension containing magnetic MOFs under vigorous stirring and kept for 5 h under mechanical agitation. Then, the solution was treated with N_2_, and a small amount of CH_3_CH_2_(OH)CH_3_ was added. The above mixing solution, sealed in a glass bottle, was placed in the radiation equipment, and irradiated using 60-Co γ-rays. After irradiation, the resulting products were washed and dried for 12 h. As shown in Figure 8b,c, the surface of Fe_3_O_4_@MIL-100(Fe) was successfully synthesized.

#### 2.2.2. Combining Functional Materials

##### Combining GO Material

Graphene oxide (GO) coupled with inorganic–organic hybrid materials, from 0D coordination complexes to 3D MOFs, offers promising prospects. The synergic effects of these two materials can lead to enhanced or even new properties [77].

**Figure 9:**
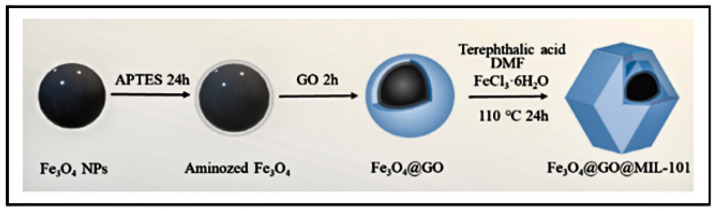
The fabrication strategy of Fe_3_O_4_@GO@MIL-101(Fe) [78]. Adapted with permission from Ref. [78]. Copyright 2024, copy-right Lin He.

Liu et al. [11] successfully synthesized GO/MIL-101(Fe). To prepare GO/MIL-101(Fe), terephthalic acid and FeCl_3_·6H_2_O were dissolved in DMF and stirred at room temperature for 1 h. Then, a certain amount of GO was added to ethanol and ultrasonically dispersed, later added to the above solution. The mixed solution was regularly sonicated until the two solutions were completely mixed. Later, the mixture was placed in a reaction kettle and reacted at a constant temperature of 120 °C for 24 h. After the reaction kettle had cooled down to room temperature, the obtained sample was centrifuged. He et al. [78] added FeCl_3_·6H_2_O and terephthalic acid into DMF and stirred them to obtain a dispersed solution (as shown in Figure 9). Then, Fe_3_O_4_@GO powder was added to the solution. The solution was transferred to a reactor and reacted for 24 h. After cooling, the solid was collected and washed alternately. Finally, brown Fe_3_O_4_@GO@MIL-101 was obtained.

**Figure 10:**
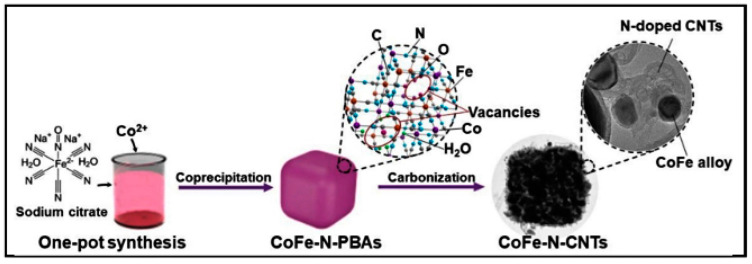
The schematic diagram of the preparation of CoFe-N-CNTs [79]. Adapted with permission from Ref. [79]. Copyright 2020, copy-right Zhen Wang.

According to Figure 10, Wang et al. [79] successfully modified MOFs with carbon nanotubes. In the process, Co(NO)_3_·6H_2_O, sodium citrate, and Na_2_[Fe(CN)_5_NO]·2H_2_O were mixed in ultrapure water and incubated at room temperature. The sediment was washed with ultrapure water, collected by centrifugation, and vacuum-dried. The materials were labeled as CoFe_m_-N-PBAs (where m represents the Fe/Co mole ratio). Finally, CoFe_m_-N-PBAs were annealed to obtain CoFe_m_-N-CNTs.

##### Combining Biomass

Biomass, an abundant, inexpensive, renewable nature material is a potential material for modifying MOFs [80]. Benefitted by its porous network, large surface area, and abundant surface functional groups that facilitate its coordination with the metal centers of MOF materials, cellulose-rich biomass-derived biochar can be considered as a good candidate for the immobilization of MOF materials [81].

**Figure 11:**
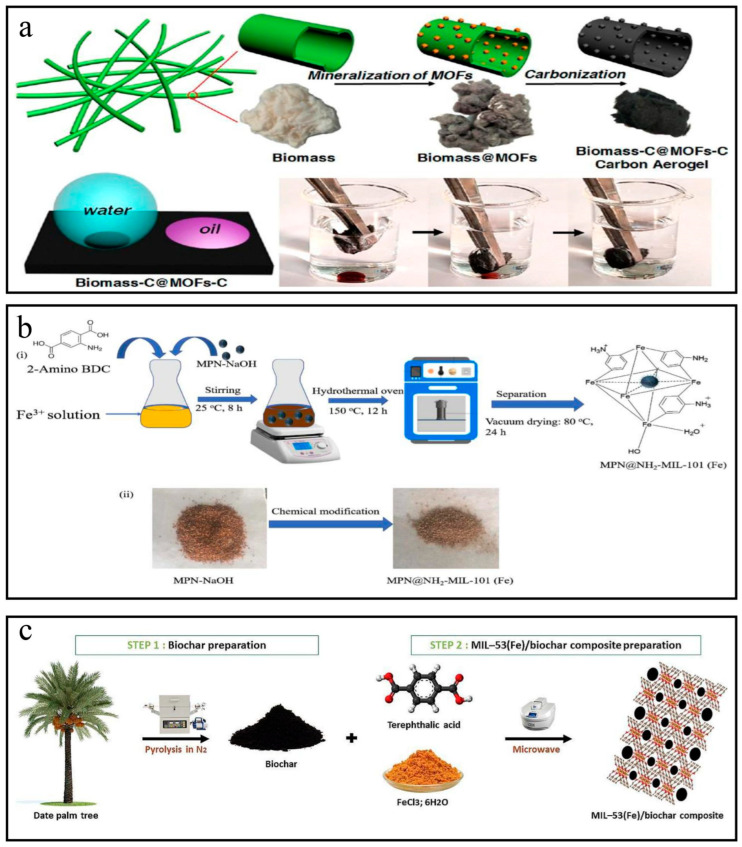
(**a**) The schematic preparation of kapok biomass-C@MOFs-C [82]. (**b**) The schematic diagram showing the synthetic route for MPN@NH_2_-MIL-101(Fe) (ⅰ) is the steps of the experiment and (ⅱ) is the figures of reactants and products [83]. (**c**) The schematic preparation of palm tree biochar MOF [81]. Adapted with permission from Ref. [82]. Copyright 2020, copy-right Yang Zhao. Adapted with permission from Ref. [83]. Copyright 2023, copy-right Aaron Albert Aryee. Adapted with permission from Ref. [81]. Copyright 2023, copy-right Hanane Chakhtouna.

Zhao et al. [82] put kapok fibers into a solution containing Fe^3+^ ions, which could be absorbed on the surface of the kapok fibers through electronic interaction. As shown in Figure 11a, after methanol washing, the kapok fibers loaded with Fe^3+^ were transferred to the methanol solution containing the organic ligand terephthalic acid, where it came into contact with the Fe^3+^ adsorbed on the surface of kapok fibers, resulting in the nucleation and growth of MIL-53(Fe) nanoparticles. As shown in Figure 11b, Aaron Albert Aryee et al. [83] mixed the magnetized peanut husk (MPN-NaOH) in a solution dissolving about FeCl_3_·6H_2_O and amino-terephthalic acid. The mixture was transferred into an autoclave and heated. Then, the material was washed several times with methanol after drying. Figure 11c illustrated that Chakhtouna et al. [81] used palm tree biochar to modify MOFs. The first step involved the preparation of biochar. In short, dates were collected, washed to remove surface dirt, cut into short sections, and dried. The resulting dried inflorescences were crushed into a fine powder and pyrolyzed under continuous N_2_ gas flow. After the pyrolysis process was completed, the BDPR-labeled biochar was washed with ultrapure water, dried, and stored for subsequent use. The second step involved the modification of the MOF. Briefly, they sonicated the biochar previously prepared from date palm trees with DMF. The mixture was then placed into a cylindrical heat-resistant glass microwave vial to which Fe(III) chloride hexahydrate and terephthalic acid were added. The flask was placed in a microwave reactor and microwave-irradiated with magnetic stirring following gradient programming. Once the reaction was complete, the solids were separated by centrifugation and then washed with absolute ethanol until the supernatant was clear to remove residual trace amounts of DMF. The collected powder was vacuum-dried to obtain a final product labeled MIL-BDPR.

#### 2.2.3. Molecular Imprinting Technology (MIT)

Molecular imprinting is an efficient technique used to prepare molecularly imprinted polymers (MIPs) that generate specific recognition sites, complementary to template molecules in shapes, sizes, and functional groups. It features the ability to selectively separate template molecules through sorption [84].

MIPs are polymers with high affinity and specific recognition sites obtained through MIT. These polymers are prepared by the polymerization of functional monomers and crosslinkers in the presence of template molecules, resulting in complementary cavities matching the shape, size, and chemical function of the template molecules [85]. According these advantages, MIPs are widely used in drugs [86,87], food [88,89], and the environment [90,91].

**Figure 12:**
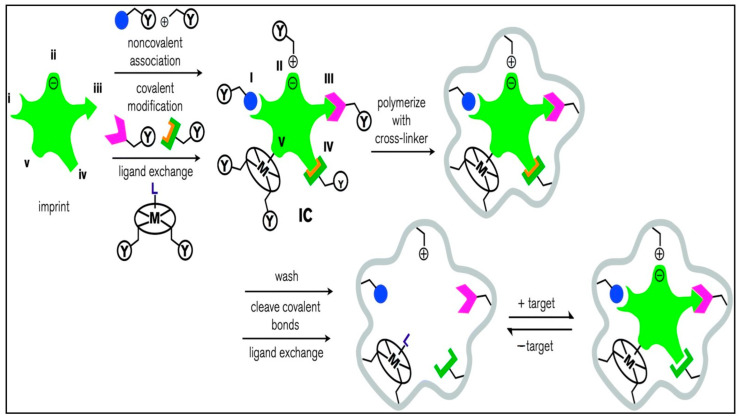
The process of molecular imprinting [92]. Adapted with permission from Ref. [92]. Copyright 2014, copy-right Jennifer E. Lofgreen.

There are five primary forms of molecular imprinting in Figure 12: metal central coordination (V), covalent, semicovalent, electrostatic/ionic (II), and noncovalent (I). By noncovalent, covalent, or ligand-to-metal (L) interactions with complementary functional groups on the imprint, an imprint molecule is joined with a suitably selected functional monomer. The functional monomer is bound to the imprint molecule (I) through hydrogen bonding or van der Waals interactions; (II) through electrostatic or ionic interactions (the charges on the imprint and functional monomer may be reversed); (III) through a covalent bond; (IV) through a covalent bond with an orange spacer; or (V) through ligand–metal or metal–ligand coordination. This results in the formation of a complex between the imprint and functional monomer (IC). Y, a functional group present in the functional monomer, engages in a cross-linking reaction with the suitable cross-linker. The imprint functional monomer connections remain intact after polymerization of the complex with a cross-linker to create the solid polymer matrix (gray). By washing, breaking chemical connections, or exchanging ligands, the imprint is eliminated, leaving an imprint hole with functional groups on the walls in its place. Target molecules that fit into the cavity and have the right structure are then taken up by noncovalent interactions (in types I, II, and IV), covalent bond formation (in type III), or ligand exchange (in type V). The matrix may also participate in target recognition and binding through nonspecific surface interactions that result from surface features created around the imprint molecule during cross-linking [92].

Recently, several studies have reported the binding of MIPs to MOFs, so that specific recognition sites and abundant porosity for selective adsorption and a large specific surface area for effective adsorption can be obtained simultaneously [93]. Habibeh Eskandari et al. [94] reported MIP/MOF-76 for targeted detection of cefixime. Jiang et al. [95] successfully managed to combine MIP and Zr-MOF UIO-66-NH_2_ for practical detoxification of organophosphorus nerve agents.

**Figure 13:**
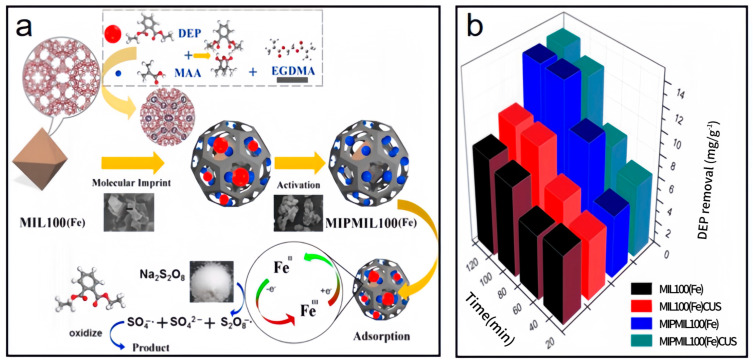
(**a**). The schematic preparation of MIPMIL100(Fe) [96]. (**b**) The degradation of DEP from materials after adsorption [96]. Adapted with permission from Ref. [96]. Copyright 2020, copy-right Xitong Li.

Li et al. [96] successfully prepared MIPMIL100(Fe), following the steps of Figure 13a. In brief, diethyl phthalate (DEP) was added to chloroform. Then, methacrylic acid and MIL-100(Fe) were added to the solution while stirring. Finally, ethylene dimethacrylate and 2-methylpropionitrile were dissolved in the solution. Under the protection of ethanol, the mixture reacted in a glass mold. Before Soxhlet extraction, the powder was washed with deionized water and ethanol. Figure 13b shows the removal efficiency of DEP after adsorption and exposure to the PS catalyst. The results showed that the maximum value of each material was reached after 90 min, and the removal rate of DEP by MIL100(Fe) was 9.62 mg·g^−1^. MIPMIL100(Fe) had the best removal efficiency of 13.64 mg·g^−1^.

We have summarized the above methods and tabulated their advantages and disadvantages in Table 3 above. In the following, we will specify the advantages and disadvantages of these modification methods.

Combining functional materials is beneficial to improve the performance of Fe-MOFs, such as enhancing the catalytic efficiency and improving the selectivity. Combining with GO is one of the most common modification methods. Modifications using GO have attracted the attention of many scholars due to the fact that the dense array of atoms and oxygen functional groups (e.g., carboxyl, epoxide, and hydroxyl) of GO can effectively increase the coordination sites of Fe-MOFs, improve chemical stability and thermal stability. However, doping GO can cause processing problems such as dust and clustering during the reaction. It also requires an increase in reaction pressure, which could lead to crystal fracture and a loss of properties of Fe-MOFs [97]. Ion doping can effectively fuse the properties of different ions into Fe-MOFs, which helps to expand the application range of Fe-MOFs. Ion doping can effectively catalyze the activation of PS and enhance the ability of Fe-MOFs to degrade pollutants. Different ion doping can provide different effects, such as increasing active sites, increasing water stability, solvent molecular sensing ability, increasing or decreasing the specific surface area, etc. [98]. However, different ion doping into Fe-MOFs requires different conditions, which leads to slow progress in ion doping technology and complex research. The arrangement of cellulose in biomass affects the electrical conductivity [99], improves light responsiveness, enhances photovoltaic conversion [100], and strengthens the activation PS capacity [83]. However, biomass doping in Fe-MOFs leads to its thermal stability degradation, structural fragility, and many other problems, which are yet to be improved. Molecular imprinting technology has specific recognition, selective adsorption, and targeted degradation. Molecularly imprinted polymers (MIPs) are designed to create customized molecular recognition sites that match the shape, size, and functional groups of the template molecule, enabling selective binding and recognition. The feasibility of MIPs in combination with other environmentally functional materials for selective removal of target contaminants has been demonstrated [101]. Currently, molecular imprinting technology is very mature, but molecular imprinting combined with Fe-MOFs is less studied, and the combination of the two is not very mature and remains to be considered in the future.

## 3. Removal Performance of Pollutants

### 3.1. Removal Antibiotics

The mass production and use of antibiotics have saved countless humans infected by bacteria. Antibiotics have been applied in clinical, agricultural, aquaculture, animal husbandry, food processing, and other fields, making significant contributions to disease prevention and control, as well as promoting the growth of animals and plants [102]. According to their mechanism of action, antibiotics mainly include aminoglycosides, tetracyclines (TCs), sulfonamides (SAs), fluoroquinolones, macrolides, etc. [103].

#### 3.1.1. Removal of Sulfonamides

Sulfonamides are the first systemic effective chemotherapeutic agents used to prevent and treat bacterial infections in humans and animals, and they are structural analogues of para-aminobenzoic acid [104]. Sulfonamides contain a large number of congeners such as sulfadimethazine, sulfadiazine (SDZ), sulfathiazole, sulfamethoxazole (SMX), etc. The overuse of sulfonamides, especially in the livestock industry, increases the potential contamination of sulfonamides in water and soil environments. Most sulfonamides are partially excreted from human and animal organisms in an unmetabolized form [105].

**Figure 14:**
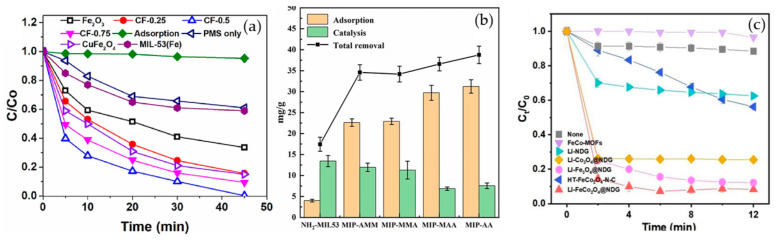
[106] (**a**) The catalytic oxidation of SMX under different catalytic systems. [107] (**b**) The selective adsorption and catalysis performance of NH_2_-MIL-53(Fe) and a series of MIP materials. [108] (**c**) Various catalysts in PMS activation for SMX degradation. Adapted with permission from Ref. [106]. Copyright 2023, copy-right Abdul Hannan Asif. Adapted with permission from Ref. [107]. Copyright 2022, copy-right Yongchang Xie. Adapted with permission from Ref. [108]. Copyright 2023, copy-right Liqin Chen.

Abdul Hannan Asif et al. [106] reported a kind of Fe-MOF for dealing with it. As shown in Figure 14a, a 38% SMX removal rate was recorded by PMS alone. In addition, the MIL-53(Fe)/PMS catalytic system only removed about 40% of SMX, and its catalytic effect was limited. Thus, Xie et al. [107] used molecular imprinting technology to improve the catalytic performance of Fe-MOFs. According to Figure 14b, NH_2_-MIL-53(Fe) had almost no adsorption capacity for SMX, and it was only about 4 mg/g after adsorption for 24 h, indicating that the adsorption performance of molecularly imprinted MOFs was significantly improved. In addition to this, the total removal reached more than 34 mg/g in the MIP system through the synergistic adsorption and catalytic action of the molecular imprinting material, and the degradation rate of NH_2_-MIL-53 (Fe) reached more than 34 mg/g, which was better than that of NH_2_-MIL-53 (Fe) at 17.44 mg/g. Chen et al. [108] proposed to use ion modification to improve the catalytic capacity of Fe-MOFs. The catalytic capacity of pure Fe-Co-MOFs for PMS was low, and the degradation rate of SMX was nearly zero within 12 min, according to the Figure 14c. Due to the introduction of metals, the activity of the catalyst was further improved. When Fe-Co-MOFs was combined with monometallic iron or cobalt oxides (LI-Fe_3_O_4_@nitrogen-doped graphene-wrapped (NDG) and LI-Co_3_O_4_@NDG), the degradation efficiency of SMX increased to 87.8% and 74.5%, respectively, under the same conditions. LI-FeCo_2_O_4_@NDG increased the degradation efficiency of SMX to 92.2% in just 6 min.

In addition to the afore mentioned Fe-MOFs, we have also collected a portion of other Fe-MOFs for the treatment of SMX in the following Table 4.

#### 3.1.2. Removal of Tetracycline

TC acts as an important broad-spectrum antimicrobial agent to achieve bactericidal effects by interfering with bacterial protein synthesis, such as Gramella and protozoan parasites, to control and treat diseases. Due to its special advantages such as broad spectrum, low toxicity, and low cost, it has been widely used in human medicine, animal husbandry, and other fields [116]. However, TC is metabolized or poorly absorbed in the animal’s digestive tract, and most of the unmetabolized form is released through excretion. As a result, it can enter the environment through animal feces and urine and is ubiquitous in surface water, groundwater, and soil [117].

**Figure 15:**
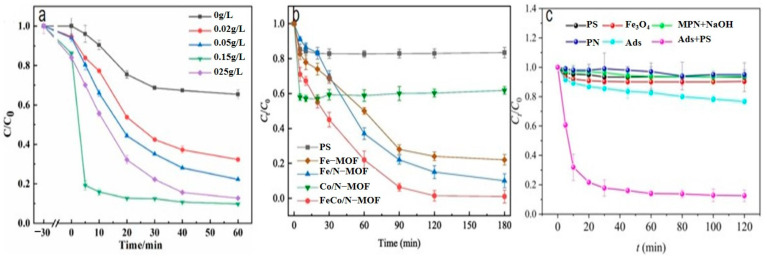
(**a**) Catalyst dosage effects of the degradation system on the TC degradation efficiency [118]. (**b**) The degradation of TC by PS only, Fe-MOF/PS, Fe/N-MOF/PS, Co/N-MOF/PS and FeCo/N-MOF (7:3)/PS [119]. (**c**). The comparison of different systems for TC removal [83]. Adapted with permission from Ref. [118]. Copyright 2023, copy-right Xiaoxiao Xie. Adapted with permission from Ref. [119]. Copyright 2022, copy-right Yifei Zhang. Adapted with permission from Ref. [83]. Copyright 2023, copy-right Aaron Albert Aryee.

Xie et al. [118] reported a nitrogen-doped catalyst (Fe-NPC) of MOFs for the degradation of TC. The degradation rate of TC was significantly enhanced at a catalyst dose of 0.02 g/L, as demonstrated by Figure 15a. This improvement was attributed to the availability of additional active sites for TC degradation at higher catalyst dosages. The findings demonstrated that at a dosage of 0.15 g/L, the degradation rate could reach 82% in under 5 min. Zhang et al. [119] used bimetals for improved catalytic performance. In the case of PS alone, the removal rate of TC after reaction equilibrium was only 17%, indicating that the oxidation capacity of persulfate to decompose a lot of pollutants at room temperature and pressure was insufficient. Specifically, as shown in Figure 15b, the catalytic activities followed the order Co/N-MOF < Fe-MOF < Fe/N-MOF < FeCo/N-MOF, and the corresponding TC removal efficiencies were 40.21%, 77.56%, 85.33%, and 98.60%, respectively. In order to confirm the efficiency of MPN@NH_2_-MIL-101(Fe)-activated persulfate for TC degradation, Aaron Albert Aryee et al. [83] compared its degradation efficiency with some of its individual components. Based on Figure 15c, using MPN@NH_2_-MIL-101(Fe) as an adsorbent (labeled as Ads) showed some removal efficiency (i.e., 23.4%). However, with the addition of persulfate (PS), the degradation efficiency of MPN@NH_2_-MIL-101(Fe) reached 87%. This may be attributed to its ability to generate free radicals from persulfate, thus confirming the feasibility of MPN@NH_2_-MIL-101(Fe) as an activator of persulfate in generating sulfate radicals.

In addition to the afore mentioned Fe-MOFs, we have also collected a portion of other Fe-MOFs for the treatment of TC in the following Table 5.

**Figure 16:**
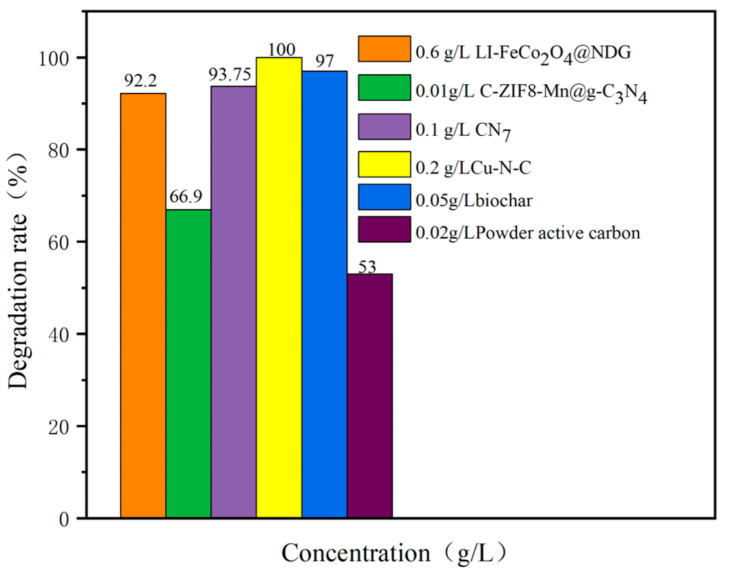
Degradation efficiency of TC by different catalytic materials.

We summarized some of the materials from the literature researched and placed them in Figure 16 for comparison. We found that materials doped with elemental carbon and nitrogen generally had a higher catalytic efficiency. This is supposedly due to the fact that carbon and nitrogen provide materials with more active sites and enhance the adsorption capacity of materials.

### 3.2. Removal of Organic Dyes

Organic dyes are commonly used color additives, widely employed in industry, scientific research, and various aspects of daily life. According to data, global organic dye production reaches 700,000 tons per year, with nearly 10–15% being discharged into industrial and domestic wastewater [122]. This has become a significant source of water pollution, posing a threat to the ecological environment and public health.

**Figure 17:**
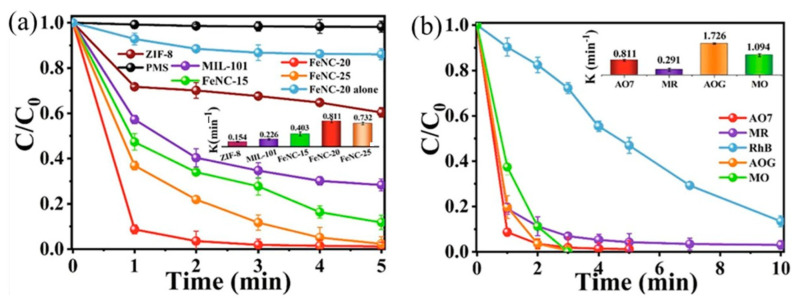
(**a**) Degradation of AO7 by PMS with various catalysts. (**b**) Degradation of various organic dyes by FeNC-20/PMS [124]. Adapted with permission from Ref. [124]. Copyright 2022, copy-right Meng Li.

AO7 is an azo dye commonly used in the dyeing and direct printing industries. The cleavage of the azo bond in AO7 produces aromatic amines, which are considered mutagenic and carcinogenic, making it an environmental pollutant [123]. Li et al. [124] discovered that FeN_4_-doped carbon nanotubes demonstrated exceptional degradation of AO7 when peroxymonosulfate (PMS) was present. The degradation of AO7 was significantly improved in the FeN4/PMS system compared to that in MIL-101(Fe)/PMS (Figure 17a). AO7 exhibited the highest degradation efficiency among the various dyes in the FeN_4_/PMS system (Figure 17b).

**Figure 18:**
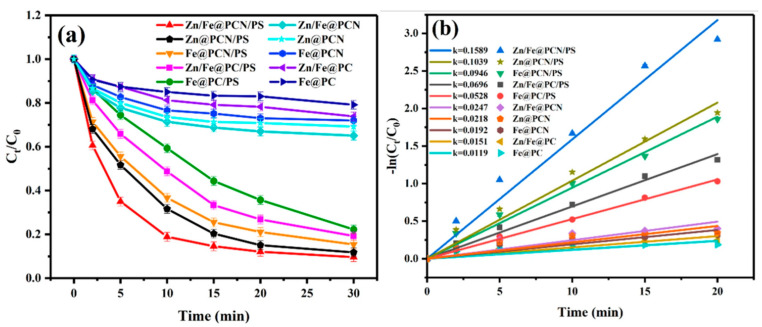
(**a**) Different systems for the degradation of RhB. (**b**) Kinetic analysis [125]. Adapted with permission from Ref. [125]. Copyright 2023, copy-right Dengjie Zhong.

RhB is a synthetic dye commonly used in the textile industry due to its stability. In order to analyze the activation properties of the Zn/Fe@N-doped porous graphitic carbon catalyst (Zn/Fe@PCN) on persulfate (PS), Zhong [125] compared the degradation of RhB in different systems, according to Figure 18. The study found that the degradation rate of RhB could be effectively enhanced through Zn and Fe doping [126]. This enhancement was attributed to the synergistic effect of Zn and Fe, as well as the presence of ZnO, which promoted the reduction of Fe^2+^ and activated PS.

**Figure 19:**
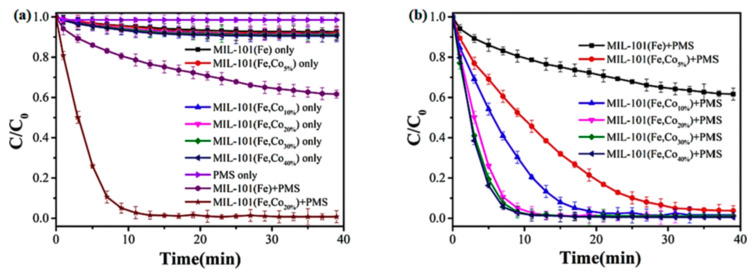
(**a**,**b**) The degradation of RhB under different reaction conditions [127]. Adapted with permission from Ref. [127]. Copyright 2023, copy-right Ziyi Xiao.

During the adsorption experiment, the concentration of RhB in Figure 19a hardly changed, indicating that MIL-101(Fe) and cobalt-doped MIL-101(Fe, Co) had almost no adsorption effect on RhB. Apparently, PMS alone had almost no oxidation effect on RhB. However, the removal of RhB by MIL-101(Fe) and MIL-101(Fe, Co) doped with different proportions of cobalt was significantly enhanced after the addition of PMS, indicating their ability to undergo Fenton-like reactions. Compared to the MIL-101(Fe)/PMS system (38%), the MIL-101(Fe, Co)/PMS system (higher than 97%) doped with different proportions of cobalt showed a higher RhB removal efficiency, as shown in Figure 19b, confirming that there was a clear synergy between Fe and Co sites. The degradation efficiency of RhB gradually increased as the Co content increased from 5% to 20%. However, the degradation efficiency of RhB did not change much when the proportion of Co increased from 20% to 40%, and the high proportion of Co doping may also cause more leaching of Co^2+^ to pollute the aquatic environment [127].

In addition to the afore mentioned Fe-MOFs, we have also collected a portion of other Fe-MOFs for the treatment of organic dyes in the following Table 6.

### 3.3. Removal of Phenols

Phenolic compounds are considered to be one of the most serious contributors to water pollution due to their high toxicity and carcinogenicity. These compounds are mainly produced by different industrial processes and are often discharged into the environment without proper treatment. Due to the widespread use of phenolic substances in modern industries such as polymer resins, coatings, petroleum, petrochemicals, etc., they are prevalent in environments that cause serious water pollution [129].

**Figure 20:**
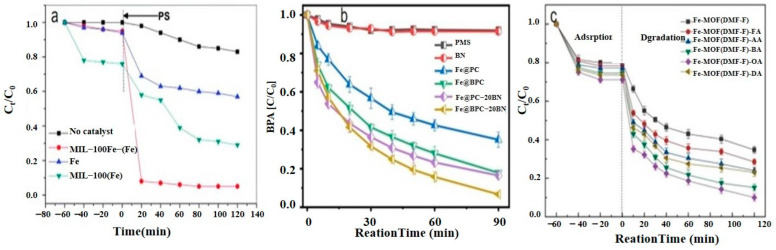
(**a**) Degradation of BPA with different catalytic conditions [6]. (**b**) Removal efficiency of BPA in different systems [130]. (**c**) Removal of TBBPA with different catalysts [131]. Adapted with permission from Ref. [6]. Copyright 2018, copy-right Yu Wang. Adapted with permission from Ref. [130]. Copyright 2022, copy-right Yantao Wan. Adapted with permission from Ref. [131]. Copyright 2020, copy-right Mei Huang.

Wang et al. [6] analyzed the degradation of BPA, as shown in Figure 20a. The figure illustrates the time dependence of BPA removal on different catalysts. The resulting data indicated that the adsorption of BPA on the above materials was a relatively short-lived process, reaching adsorption/desorption equilibrium at around 40 min. When persulfate was further introduced into the system, a dramatic increase in BPA degradation was subsequently observed. In the MIL-101(Fe)-Fc/PS process, BPA was almost completely removed in 40 min. Wan et al. [130] evaluated the degradation performance of the B,N-decorated carbon catalyst (Fe@BPC-XBN) using BPA as the target pollutant. According to Figure 20b, the removal rate of BPA by Fe@BPC-20BN alone was only 4.3%, indicating that the contribution of adsorption to the removal of BPA was also not significant. PMS activation using Fe@PC and Fe@BPC degraded about 65% and 82% of BPA, respectively, confirming the good catalytic ability of B doping alone on carbon catalysts for PMS activation. In addition, the activation of PMS using Fe@PC-20BN and Fe@BPC-20BN degraded about 83.5% and 93.3% of BPA, respectively, compared with Fe@PC and Fe@BPC, indicating that the addition of BN during the synthesis of carbon catalysts can further improve the removal of BPA. Huang et al. [131] introduced different modulators with different lengths to prepare defective Fe(BDC)(DMF, F)-X. As shown in Figure 20c, the adsorptive removal of TBBPA reached 20.1%, 21.6%, 22.8%, 25.3%, 28.9%, and 26.4% for all materials within 60 min, respectively. Once the persulfate was introduced, a sharp increase in TBBPA removal was observed, indicating that the catalyst could effectively activate the persulfate to produce the initial ^•^OH and/or SO_4_^•−^, bringing about the oxidative degradation of the target pollutants. Among them, the addition of Fe(BDC)(DMF, F)-octanoic acid (OA) reached a TBBPA removal rate of 90.13% in 120 min. Meanwhile, the TBBPA removal rates of other catalyst/PS systems reached 65.25%, 71.49%, 75.92%, 84.91%, and 76.87%, respectively.

In addition to the afore mentioned Fe-MOFs, we have also collected a portion of other Fe-MOFs for the treatment of Phenols in the following Table 7.

### 3.4. The Comparison of Fe-MOFs with Other MOFs, and Materials

**Figure 21:**
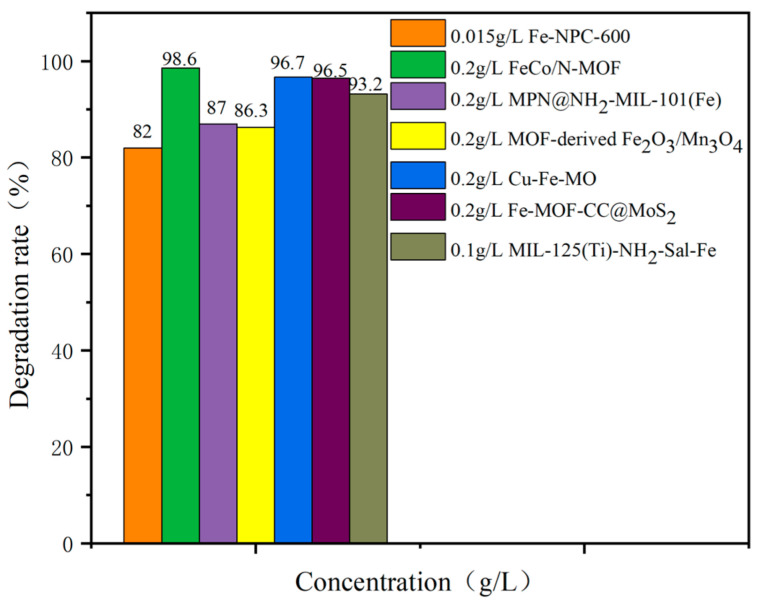
Degradation efficiency of SMX by different catalytic materials.

According to Table 8 and Figure 21, our Fe-MOFs have very good results in degrading SMX, but they are still insufficient compared with some materials. This may be because of the insufficiency of our literature search. Overall, Fe-MOFs still have great potential in degrading pollutants and have the advantages of high reusability and a fast reaction time. As can be seen in Table 8 and Figure 21, carbon alone is not very efficient in removing SMX, but in combination with other materials, the catalytic efficiency increases significantly. Therefore, we mentioned earlier in the article that the modification of Fe-MOFs using GO helped to improve the catalytic efficiency of the catalyst. It is also mentioned in Table 8 and Figure 21 that the copper monometal doped nitrogen and carbon catalyst has the highest catalytic efficiency. This is achieved precisely by utilizing the combined functional materials we mentioned above. Carbon and nitrogen are the most common doping elements. These two elements, with large reserves, easy accessibility, many active sites, and structural stability after binding, are ideal binding materials. It is easy to see in Table 8 that biomass materials also play an important role in enhancing the efficiency of the catalyst. This is because a biomass material has a strong adsorption capacity, which can increase the contact time between the catalyst and the reactants and the amount of contact, so as to achieve the effect of accelerating the reaction, and at the same time, there are also some reactants adsorbed by the biomass material to remove, which further improves the degradation rate. Of course, our summary is one-sided, summarizing only the role of different catalysts in degrading SMX and not comparing against other contaminants.

## 4. Degradation Mechanisms of Fe-MOFs/PS

Currently, two primary degradation mechanisms for Fe-based MOFs are recognized, the non-free radical (^1^O_2_) pathway and the free radical (•OH, O_2_^•−^ and SO_4_^•−^) pathway in PAOT. The quenching experiment [139] and electron paramagnetic resonance (EPR) characterization are established procedures for identifying free radicals and non-free radicals.

The quenching experiment involves adding specific compounds to the reaction system that exhibit a high reaction rate with specific radicals, resulting in the rapid consumption of the radicals in the system [140]. Common free radical quenchers include methanol (MeOH), tert-butanol (TBA) and p-benzoquinone (pBQ). A common non-free radical quencher is L-histidine. MeOH scavenges SO_4_^•−^ and •OH radicals, TBA scavenges •OH, and pBQ scavenges O_2_^•−^. L-histidine scavenges ^1^O_2_. EPR is the direct determination of free radicals and non-free radicals by identifying spectral properties [141]. Common spin-trapping agents are 5,5-dimethyl-1-pyrroline N-oxide (DMPO) and 2,2,6,6-Tetramethyl-4-piperidone hydrochloride (TEPM). DMPO is used as the spin-trapping agent for free radicals. TEMP is used as the spin-trapping agent for ^1^O_2_.

### 4.1. Radical Pathway

**Figure 22:**
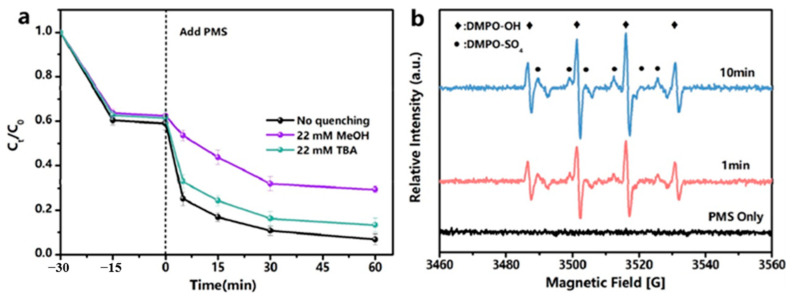
(**a**) Effect of different radical quenchers on TC degradation in Mn-MIL-53(Fe)/PMS system. (**b**) EPR spectra obtained by using DMPO as spin-trapping agent [142]. Adapted with permission from Ref. [142]. Copyright 2022, copy-right Jun Yu.

Yu et al. [142] synthesized manganese-doped MIL-53(Fe) via a one-pot thermal method and employed it to activate PMS for degrading TC. The results from the free radical scavenging experiments showed that both TBA and MeOH inhibited the degradation of TC but to varying degrees. The addition of TBA resulted in a slight decrease in the removal of TC from 93.2% to 86.7%, while the addition of MeOH decreased the removal to 70.6%, as shown in Figure 22a. The results suggested that MeOH strongly inhibited the reaction, with SO_4_^•−^ being the dominant species. An EPR analysis confirmed the presence of characteristic signals of DMPO-OH and DMPO-SO_4_, supporting the results of the free radical scavenging experiments. Furthermore, the intensity of both characteristic signals increased as the time was extended from 1 to 10 min, as shown in Figure 22b. This indicates that Mn-MIL-53(Fe) activated PMS, resulting in the generation of more SO_4_^•−^ and •OH to participate in the catalytic reaction. As a result, SO_4_^•−^ and •OH play a dominant role in the degradation of TC in the Mn-MIL-53(Fe)/PMS system.

**Figure 23:**
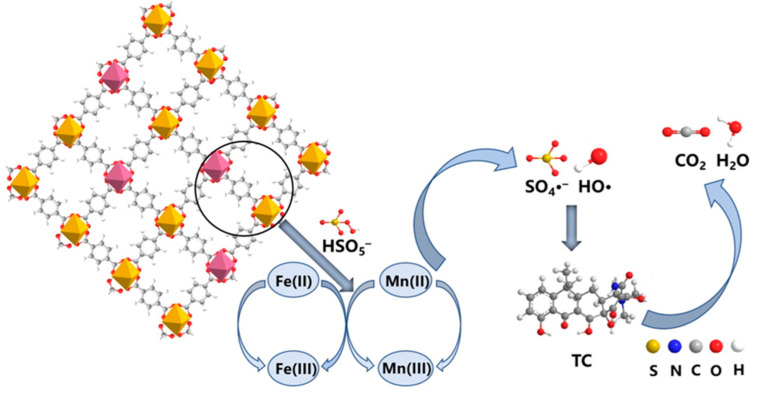
Possible reaction mechanism of TC degradation by Mn-MIL-53(Fe)/PMS system [142].

Initially, the Fe(II) and Mn(II) sites on the catalysts generated SO_4_^•−^ and ^•^OH by providing electrons to activate PMS. Fe(II) and Mn(II) also transformed into Mn(III) and Fe(III) after losing electrons (Equations (1)–(4)). Subsequently, the Mn(III) and Fe(III) sites received electrons to activate PMS, generating SO_4_^•−^ radicals and the sites of Mn(II) and Fe(II), forming a cycle of Mn and Fe (Equations (5)–(7)). Finally, the generated SO_4_^•−^ and •OH radicals could efficiently degrade TC molecules. The catalytic performance of Mn-MIL-53(Fe) was superior to that of MIL-53(Fe), which might be due to the synergistic effect that could occur between Mn and Fe. Furthermore, the electron transfer rate could be enhanced due to the redox reaction occurring between Mn and Fe, improving the activity of SO_4_^•−^ and •OH produced by PMS. Both factors improved the degradation efficiency and reusability of the catalysts. The possible mechanism is shown in Figure 23 [142].

(1) $$Mn(II)+HS{O_{5}}^{-}\to Mn(III)+S{O_{4}}^{2-}+•OH$$

(2) $$Fe(II)+HS{O_{5}}^{-}\to Fe(III)+S{O_{4}}^{2-}+•OH$$

(3) $$Mn(II)+HS{O_{5}}^{-}\to Mn(III)+S{O_{4}}^{2-}+OH^{-}$$

(4) $$Fe(II)+HSO_{5}{\;}^{-}\to Fe(III)+SO_{4}{\;}^{2-}+OH^{-}$$

(5) $$Mn(III)+HSO_{5}{\;}^{-}\to Mn(II)+SO_{5}{•}^{-}+H^{+}$$

(6) $$Fe(III)+HSO_{5}{\;}^{-}\to Fe(II)+SO_{5}{•}^{-}+H^{+}$$

(7) Mn(III)+Fe(II)→Mn(II)+Fe(III)

### 4.2. Non-Radical Pathways

Pu et al. [21] investigated the reaction mechanism of Fe@C-800/PS by performing molecular probe experiments to identify the reaction intermediates produced. The results of quenching experiments showed that the addition of MeOH and TBA had a significant inhibitory effect on the degradation of SMX. From the result shown in Figure 22a, the degradation rate was drastically reduced by 53% and 68.6%, respectively, suggesting that SO_4_^•−^ and ^•^OH were partially responsible for the degradation of SMX. However, even after 180 min, the removal of SMX was still over 70%, indicating the presence of other active substances contributing to its degradation. The addition of PBQ resulted in a 3.5% decrease in SMX removal after 180 min, suggesting that O_2_^•−^ also plays a role in the degradation process. The inclusion of L-histidine had a significant inhibitory effect, resulting in a 51% decrease in the degradation efficiency of SMX after 180 min. This suggests that ^1^O_2_ is the primary active substance that promotes SMX degradation.

**Figure 24:**
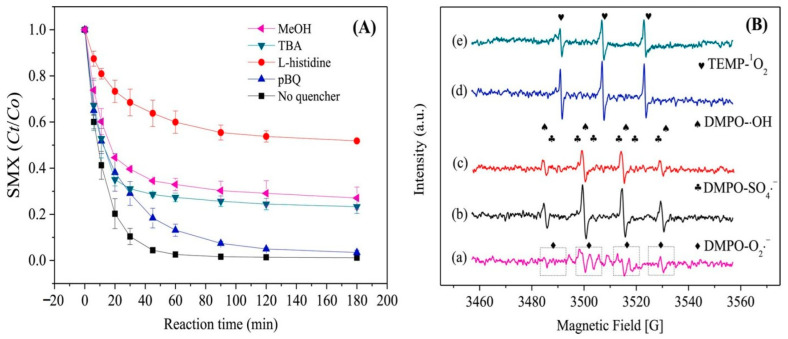
(**A**) Degradation of SMX in the presence of various quenching agents; (**B**) EPR spectra recorded in different activation systems (**a**) Fe@C-800/PS/MeOH/DMPO, (**b**) Fe@C-800/PS/DMPO, (**c**) Fe@C-800/PS/SMX/DMPO, (**d**) Fe@C-800/PS/TEMP, (**e**) Fe@C-800/PS/SMX/TEMP [21]. Adapted with permission from Ref. [21]. Copyright 2021, copy-right Mengjie Pu.

Further EPR tests were conducted to confirm the presence of the active species mentioned above. In the presence of Fe@C-800, DMPO-SO_4_^•−^ showed characteristic signals, confirming the production of both SO_4_^•−^ and •OH during the activation process (Figure 24B-(c)). Upon adding SMX to the mixture, the intensities of these two signals decreased significantly, indicating that the contaminants consumed both. Additionally, from the result shown in Figure 24A, typical DMPO-O_2_^•−^ signals were recorded upon adding MeOH to Fe@C-800/PS, verifying the formation and presence of O_2_^•−^ in this system. Furthermore, signals for the TEMP-^1^O_2_ adduct were obtained when TEMP was used as a spin-trapping agent for ^1^O_2_(Figure 24B-(e)). The test signals were attenuated when SMX was present, indicating the inclusion of ^1^O_2_ as an alternative degradation pathway in this system [21].

Based on quenching experiments and EPR results, Fe^0^, Fe^2+^, and Fe^3+^ active species on the surface of Fe@C-800 could provide electrons for PS activation to generate SO_4_^•−^ or persulfate radical (S_2_O_8_^•−^) through the radical process (Equations (8)–(11)). Additionally, the hydrolysis of PS (mediated by the hydroperoxide anion (HO_2_^−^)) and SO_4_^•−^could result in the formation of SO_4_^•−^, O_2_^•−^, and •OH (Equations (12)–(14)). Regarding the non-radical part, the direct oxidation or recombination of O_2_^•−^ (with either •OH, H^+^, H_2_O, or PS) may result in the production of ^1^O_2_(Equations (15)–(18)). However, pBQ quenching experiments suggest that this is not the primary pathway for generating ^1^O_2_. Therefore, SMX could be degraded by SO_4_^•−^, ^•^OH, O_2_^•−^ and ^1^O_2_ through radical and non-radical mechanisms in Fe@C-800/PS [21].

(8) $$Fe^{0}+2S_{2}O_{8}{\;}^{2-}\to Fe^{2+}+2SO_{4}{•}^{-}+2SO_{4}{\;}^{2-}$$

(9) $$Fe^{2+}+S_{2}O_{8}{\;}^{2-}\to Fe^{3+}+SO_{4}{•}^{-}+SO_{4}{\;}^{2-}$$

(10) $$Fe^{3+}+S_{2}O_{8}{\;}^{2-}\to Fe^{2+}+S_{2}O_{8}{•}^{-}$$

(11) $$Fe^{0}+2Fe^{3+}\to 3Fe^{2+}$$

(12) $$S_{2}O_{8}{\;}^{2-}{+2H}_{2}O\to 2SO_{4}{\;}^{2-}+HO_{2}{\;}^{-}+3H^{+}$$

(13) $$S_{2}O_{8}{\;}^{2-}+HO_{2}{\;}^{-}\to SO_{4}{\;}^{2-}+SO_{4}{•}^{-}+O_{2}{•}^{-}+H^{+}$$

(14) $$SO_{4}{•}^{-}+H_{2}O\to SO_{4}{\;}^{2-}+•OH+H^{+}$$

(15) $$O_{2}{•}^{-}+•OH\to^{1}O_{2}+OH^{-}$$

(16) $$2O_{2}{•}^{-}+2H^{+}\to^{1}O_{2}+H_{2}O_{2}$$

(17) $$2O_{2}{•}^{-}+2H_{2}O\to^{1}O_{2}+H_{2}O_{2}+2OH^{-}$$

(18) $$O_{2}{•}^{-}+S_{2}O_{8}{\;}^{2-}\to^{1}O_{2}+SO_{4}{•}^{-}+SO_{4}{\;}^{2-}$$

## 5. Conclusions

This review described the synthesis of different types of Fe-MOFs by the hydrothermal method, microwave-assisted synthesis, dry-gel conversion technology, and their excellent performance in water treatment. Although the metal centers, organic ligands, and synthesis conditions of the various Fe-MOFs are different, the types of synthesis processes all have similar characteristics. Different Fe-MOFs have the same type of synthetic method, but not all synthetic methods are suitable for the synthesis of all Fe-MOFs, despite the fact that the solvothermal method is almost suitable for most MOFs. In addition, this review summarized the removal performance and mechanism of Fe-MOFs and their derivatives for organic pollutants in water. The Fe-MOF materials remove pollutants in the aqueous environment mainly through adsorption and advanced oxidation. Fe-MOFs, with their high pore volume, excellent stability, outstanding specific surface area, abundant functional groups, and prominent active sites, have been shown to be useful in the field of water treatment to ensure the balance of the water environment. Although there have been a large number of studies on Fe-MOFs and their applications in water treatment, there are still some areas that have not been studied deeply enough or even covered. Hence, we should further study the following aspects in the future:

(1) It is necessary to find a method for the synthesis of MOFs that is green, with low energy consumption, a simple process, high crystallinity, and that can be applied to the mass production in factories. Meanwhile, finding a suitable method for large-scale synthesis in factories to realize the preparation of MOFs is also the focus of future research, since the type of instrumentation used in the experiments, experimental operation, experimental conditions, and other factors will affect the reproducibility of MOFs.

(2) Contaminants have specific physical properties, such as molecular size and functional groups. Therefore, when removing specific pollutants, Fe-MOFs with pore sizes larger than those of the pollutants have a selective ability. The combination of molecular imprinting technology and Fe-MOFs for targeted degradation of pollutants is an important direction in the future.

(3) Since Fe-MOFs are formed by combining metal ions and organic ligands, they are inherently toxic. Currently, most Fe-MOFs are degraded in laboratory-prepared aqueous solutions and are not considered for practical applications. The actual aqueous environment is much more complex, and the cost, reuse rate, and range of use need to be fully considered in practical use. Therefore, future research on practical environmental applications needs to be strengthened.

(4) Most of the pollutants were removed by adsorption of Fe-MOFs. Although there are some studies on the catalytic degradation of activated persulfate by Fe-MOFs, they are not extensive. Activated-persulfate-catalyzed removal of pollutants consumes less energy and may cause less secondary pollution. It is necessary to accelerate the research progress on the activated-persulfate-catalyzed degradation of organic pollutants by Fe-MOFs.

Based on the above problems and challenges faced by MOF materials, researchers should pay more attention to the new material synthesis methods and material modification methods, the effects of the materials themselves on the experimental results and on the natural water bodies, as well as the problems of the materials in practical applications. In conclusion, as an excellent new type of porous materials, MOFs will soon be able to reach large-scale industrialized production and practical application under the efforts of researchers.

## Figures and Tables

**Table 1:** Synthesis method, conditions, and surface area of MIL-Fe series.

Catalysts	Organic Ligands	Synthesis Methods	S_BET_ (m^2^/g)	Temperature (°C)	Ref.
MIL-53(Fe)	DMF	Solvothermal	1415	493	[38]
MIL-53(Fe)	DMF	Microwave-assisted	/	150	[48]
MIL-88(Fe)	DMF	Ultrasound-assisted	359	85	[49]
MIL-88(Fe)	H_2_O	Solvothermal	26.22	100	[50]
MIL-88(Fe)	DMF	Microwave-assisted	1242	150	[51]
MIL-88(Fe)	DMF	Solvothermal	209.83	/	[44]
MIL-100(Fe)	HF	Microwave-assisted	/	200	[52]
MIL-100(Fe)	DMF	Solvothermal	/	160	[53]
MIL-100(Fe)	HF, H_2_O	Solvothermal	1626	150	[54]
MIL-100(Fe)	HF	Solvothermal	1917	150	[55]
MIL-100(Fe)	Na_2_CO_3_	Solvothermal	1327	160	[56]
MIL-100(Fe)	DMF	Solvothermal	1501	150	[57]
MIL-100(Fe)	DMF	Radical-promoted facile	2482	25	[58]
MIL-100(Fe)	DMF	Gamma irradiation-assisted	/	180	[59]
MIL-100(Fe)	H_2_O	Dry-gel conversion	1340	165	[60]
MIL-101(Fe)	DMF	Microwave-assisted	383	110	[61]
MIL-101(Fe)	DMF	Hydrothermal	3500	/	[62]
MIL-101(Fe)	DMF	Electrochemical	/	25	[63]

**Table 2:** The advantages and disadvantages of different synthesis methods.

Method	Advantages	Disadvantages
Hydrothermal	Simple operation, easy to obtain raw materials, the most widely used, the most mature technology	Complex reaction, high risk, low efficiency, slow reaction rate, environmental concerns
Microwave-assisted	Fast reaction rate, short reaction time, uniform particle size, high yield, high phase purity	Complex operation, high technical requirements, immature technology, few relevant applications
Dry-gel conversion technology	Reduced water consumption, very mild reaction conditions, low vapor pressure and convenient	Difficult to operate, small application range, harsh conditions

**Table 3:** The advantages and disadvantages of different modifications.

Method	Advantages	Disadvantages
Combining GO material	Higher surface atomic density and enhanced surface dispersion, surface area, and porosity for enhanced selectivity and adsorption capacity	Processing issues such as dust and clustering, high compression pressures often lead to fracture or deformation of Fe-MOFs crystals and loss of properties
Ionic doping	Ionic characterization incorporated into Fe-MOFs to improve selectivity, application range, and enhance catalytic efficiency	Different reaction conditions for different ions, long preparation reaction time, few available results
Combining biomass	Environmentally friendly, strong adsorption performance, low cost, easy access to raw materials	Long pretreatment time, poor thermal stability, harsh binding conditions, fragile biomass structure
Molecular imprinting technology	Specific selection, targeted degradation, high catalytic efficiency and reusability	Difficult to synthesize, many steps, complex operation, time-consuming preparation, small application range

**Table 4:** Degradation efficiency between catalysts and sulfonamides.

Fe-MOFs	Contaminant	Structure	Degradation Rate	Ref.
MIL-53(Fe) 0.2 g/L	SMX 20 mg/L	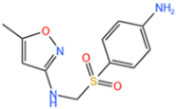	40%	[106]
NH_2_-MIL-53(Fe) 0.05 g/L	SMX 20 mg/L	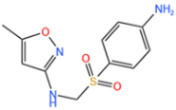	/	[107]
LI-FeCo2O4@NDG 0.6 g/L	SMX 0.5 mM	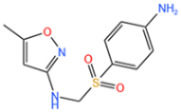	92.2%	[108]
Fe@C 0.4 g/L	SMX 10 mg/L	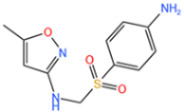	76.2%	[109]
Fe-MOFs-2 1 g/L	SMX 10 mg/L	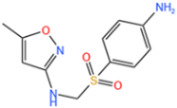	95.1%	[18]
Fe(II)MOFs 0.5 g/L	SMX 0.04 mM	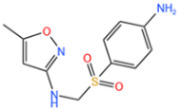	88.9%	[110]
Fe-UiO-66 0.2 g/L	SMX 10 mg	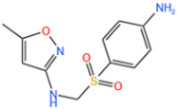	89.9%	[111]
Cu-Fe-MOF 0.2 g/L	SMX 20 mg/L	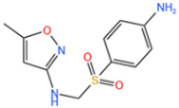	98.9%	[112]
Fe-MOF-CC@MoS_2_ 0.2 g/L	SMX 20 mg/L	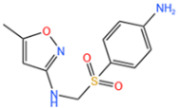	80.8%	[113]
MnS/Fe-MOF 0.2 g/L	SDZ 5 mg/L	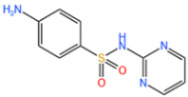	100%	[114]
Fe_3_O_4_@MoS_2_-3 0.4 g/L	SA 20 mg/L		99.8%	[115]

**Table 5:** Degradation efficiency between catalysts and TC.

Fe-MOFs	Contaminants	Structure	Degradation Rate	Ref
Fe-NPC-600 15 mg/L	TC 30 mg/L	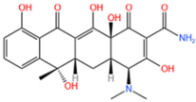	82%	[118]
FeCo/N-MOF 0.2 g/L	TC 50 mg/L	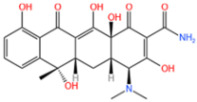	98.6%	[119]
MPN@NH_2_-MIL-101(Fe)	TC	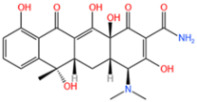	87%	[83]
MOF-derived Fe_2_O_3_/Mn_3_O_4_ 0.2 g/L	TC 10 mg/L	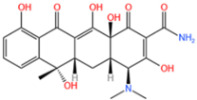	86.3%	[120]
Cu-Fe-MOF 0.2 g/L	TC 20 mg/L	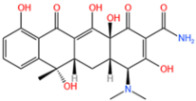	96.7%	[112]
Fe-MOF-CC@MoS_2_ 0.2 g/L	TC 20 mg/L	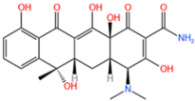	96.5%	[113]
MIL-125(Ti)-NH 2-Sal-Fe 0.1 g/L	TC 0.02 g/L	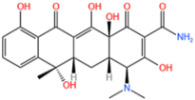	93.2%	[121]

**Table 6:** Degradation efficiency between catalysts and organic dyes.

Fe-MOFs	Contaminants	Structure	Degradation Rate	Ref.
Fe-NC nanocomposites 2 mg/L	AO7 50 mg/L	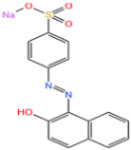	100%	[124]
Zn/Fe@PCN 0.1 g/L	RhB 50 mg/L	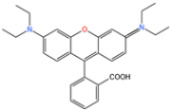	90.43%	[125]
MIL-101(Fe, Co) 0.2 g/L	RhB 10 mg/L	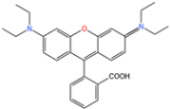	98.60%	[127]
MoS_2_/FeMoO_4_ 0.15 g/L	RhB 20 mg/L	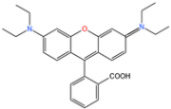	97.7%	[128]
Cu-Fe-MOF 0.2 g/L	RhB 20 mg/L	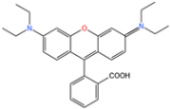	99.4%	[112]
Cu-Fe-MOF 0.2 g/L	MB 20 mg/L	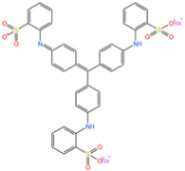	97.4%	[112]

**Table 7:** Degradation efficiency between catalysts and phenols.

Fe-MOFs	Contaminants	Structure	Degradation Rate	Ref
MIL-101(Fe) 0.2 g/L	BPA 60 mg/L	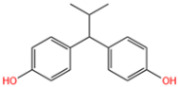	78%	[6]
Fe@BPC 150 mg/L	BPA 20 mg/L	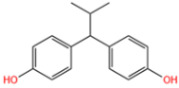	93.3%	[130]
Cu-Fe-MOF 0.2 g/L	BPA 20 mg/L	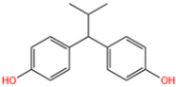	96.7%	[112]
Fe(BDC)(DMF,F)-OA	TBBPA	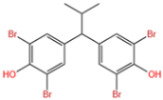	90.13%	[131]
Cu-Fe-MOF 0.5 g/L	Phenol 50 mg/L	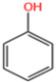	97.9%	[112]
Fe_x_C-600 0.3 g/L	Phenol 20 mg/L	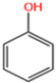	98.23%	[132]
Cu-Fe-MOF 0.5 g/L	2,4-Dichlorophenol 20 mg/L	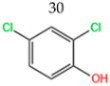	95.2%	[112]

**Table 8:** Degradation rate of different materials.

Materials	Contaminants	Structure	Degradation Rate	Ref.
LI-FeCo_2_O_4_@NDG 0.6 g/L	SMX 0.5 mM	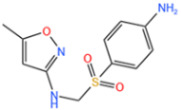	92.2%	[108]
C-ZIF8-Mn@g-C_3_N_4_ 0.01g/L	SMX 10 mg/L	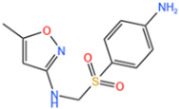	66.9%	[133]
CN7 0.1 g/L	SMX 20 mg/L	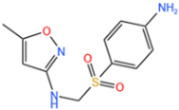	93.75%	[134]
Cu-N-C 0.2 g/L	SMX 18 mg/L	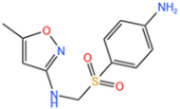	100%	[135]
KMnO_4_/biochar 100 μm/50 mg/L	SMX 10 μM	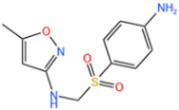	97%	[136]
Powder active carbon 0.02g/L	SMX 10 μM	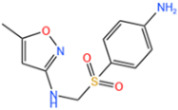	53%	[137]
Dendritic mesoporous silica-titania coupled with CuFeS_3_ 1.2g/L	SMX 10mg/L	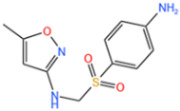	88.9%	[138]

## Data Availability

Not applicable.
